# Repetitive non-thermal melting as a timing monitor for femtosecond pump/probe X-ray experiments

**DOI:** 10.1063/4.0000020

**Published:** 2020-09-22

**Authors:** Å. U. J. Bengtsson, J. C. Ekström, Xiaocui Wang, A. Jurgilaitis, Van-Thai Pham, D. Kroon, J. Larsson

**Affiliations:** 1 Department of Physics, Lund University, P.O. Box 118, SE-221 00 Lund, Sweden; 2 MAX IV Laboratory, Lund University, P.O. Box 118, SE-221 00 Lund, Sweden

## Abstract

Time-resolved optical pump/X-ray probe experiments are often used to study structural dynamics. To ensure high temporal resolution, it is necessary to monitor the timing between the X-ray pulses and the laser pulses. The transition from a crystalline solid material to a disordered state in a non-thermal melting process can be used as a reliable timing monitor. We have performed a study of the non-thermal melting of InSb in single-shot mode, where we varied the sample temperature in order to determine the conditions required for repetitive melting. We show how experimental conditions affect the feasibility of such a timing tool.

## I. INTRODUCTION

Studies of structural dynamics are commonly performed using time resolved optical pump/X-ray probe methods.^1,2^ Many of these experiments are carried out at hard free-electron laser facilities, such as Linac Coherent Light Source (LCLS),^3^ SACLA,^4^ SwissFEL,^5^ and the European XFEL,^6^ and other short-pulse X-ray facilities such as FemtoMAX at the MAXIV Laboratory.^7^ However, combined laser and accelerator-based setups suffer from inherent temporal jitter, and the relative arrival times of the laser pulse and the X-ray pulse at the sample must be precisely determined in order to perform accurate time-dependent studies. This can be done using a number of methods.

The timing tool used at LCLS is based on the fact that when an intense X-ray pulse interacts with a material, it excites a large number of carriers, thereby modifying the transmission of a chirped visible probe. The spectral transmission can, therefore, be interpreted in terms of the difference in arrival time between the visible laser and the X-ray laser.^8^ This is a very useful device; its only drawback being that an intense X-ray pulse is required to induce the change in reflectivity. 1 × 10^10^ photons per pulse in a 0.5 mm spot gives a 10% change in the spectral encoding signal.^9^

The sub-picosecond pulse source (SPPS), which has now been decommissioned, employed a timing tool based on electro-optic sampling (EO sampling) to determine the jitter between the optical laser and vacuum ultraviolet bunches.^10^ In EO sampling, the electric field of the electron bunch introduces birefringence in an electro-optical crystal. The polarization of an optical pulse passing through the crystal will be rotated by an amount that depends on the amplitude of the electric field resulting from the electron bunch. A temporal trace can be obtained for each shot by mapping the crossed-beam geometry space onto time, thus providing a measure of the relative timing.

Two different methods for monitoring the relative timing jitter are employed at the Free-Electron Laser facility in Hamburg: one based on EO sampling and the other using a streak camera. For the second method, pulses from a bending magnet and a visible pump laser are both sent to a streak camera, where their relative arrival time is translated into a spatial difference on the camera screen. The drawback of this system is the limited temporal resolution of the streak camera.

All these above-mentioned setups measure the jitter but not the relative arrival time at the sample position. We propose an X-ray timing diagnostic tool based on non-thermal melting of InSb, which is an extensively studied ultrafast phenomenon.^11–14^ Non-thermal melting occurs when the electrons in a solid are excited into new (anti-bonding) conduction band orbitals by a laser, thus breaking the bonds between the atoms. Non-thermal melting occurs on the timescale of a few hundred femtoseconds, which is shorter than the electron–phonon-coupling time and can, thus, be distinguished from thermal melting.

As order in the solid is lost, the scattering pattern will change from that of a perfect crystal to that of a liquid. When monitoring a specific Bragg spot, the temporal overlap between the X-ray and the laser pulse will be indicated by a rapid decrease in the intensity of the diffracted X-rays. The difference in arrival time between the laser and X-ray pulses can, thus, be determined by following the X-ray signal in the temporal domain. A setup based on the non-thermal melting of InSb can be implemented so that for each X-ray/laser shot, the relative time can be determined. A timing tool based on the ultrafast melting of InSb could be implemented at a synchrotron radiation facility either as a permanent timing tool or to determine the performance of other timing tools. Non-thermal melting of InSb has been used to verify the EO sampling-based timing tool at SPPS.^10^

In order to apply non-thermal melting as a timing tool in practice, it must be possible to repeatedly melt the sample in the same position, as using a new position for each shot would increase the size of the sample required, rendering the method impractical. Under certain conditions, molten InSb will re-form into a crystal structure, enabling repeated measurements at the same position on the sample. The quality of crystal regrowth will affect the diffracted X-ray signal, and poorer quality will degrade the performance of the timing tool.

In the present study, we have investigated repetitive non-thermal melting of InSb in single-shot mode, as a means of determining the relative arrival times of the X-ray and laser pulses at the FemtoMAX beamline at MAX IV with an accuracy of 50 fs. Measurements were carried out at different temperatures between 300 and 500 K to determine the optimal conditions for repetitive melting and regrowth. The setup was also used to evaluate an RF-cavity-based timing tool currently being developed at the beamline.

## II. EXPERIMENTAL METHODS

FemtoMAX is a beamline for time-resolved X-ray studies at the MAX IV synchrotron radiation facility in Lund, Sweden.^7^ The linear accelerator provides electrons for the generation of X-ray pulses with pulse durations of <200 fs in the energy range of 1.8–20 keV. In the present experiments, an InSb double crystal monochromator with a bandwidth of <0.04% was used. The beamline has a Ti:sapphire amplifier providing femtosecond 800 nm laser pulses.

**FIG. 1.**
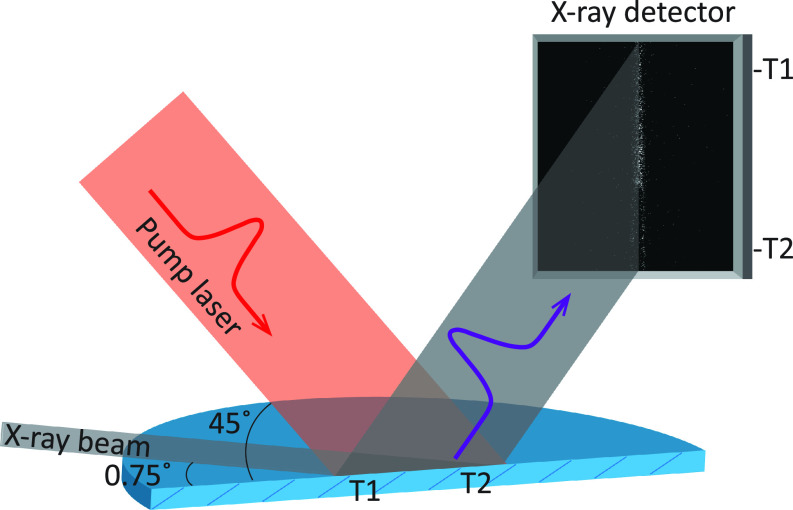
Experimental setup. The difference in the incidence angle between the pump and probe beam will map the spatial extent of the sample into a time axis.

The sample was an InSb wafer cut with the [111]-plane at an angle of 30° to the surface. The sample was pumped with the 800 nm femtosecond laser, focused in one direction using a cylindrical lens to deliver an elongated uniform laser profile matching the on-sample footprint of the X-ray probe beam. The laser fluence was <150 mJ/cm^2^, which is above the fluence threshold for non-thermal melting. The probe beam delivered 3.56 keV X-ray pulses. The X-ray incidence angle was calibrated using the specular reflection and was set to 0.75°. Figure 1 shows the experimental setup. The grazing incidence geometry was designed so that the probe depth of the X-rays (28 nm) was much smaller than the tabulated absorption depth of the laser (100 nm).^15^ The diffracted X-ray signal was captured with an Andor IKON CCD camera.

The difference in the incidence angle between the pump and probe beams allowed the change in X-ray diffraction efficiency due to laser-induced melting to be followed in time. As can be seen in Fig. 1, X-rays diffracted from different positions on the sample will be detected at different positions on the detector. The difference in the incidence angle between the pump and probe beams means that the sample position corresponding to T1 will be probed at an earlier time in the melting process than the sample position corresponding to T2. Thus, the vertical axis of the detector is mapped onto a time axis. This is seen in the signal on the detector screen where there is a clear X-ray signal at T1, when the sample has not yet been irradiated, while at T2, the X-ray signal has disappeared as a result of laser-induced melting. The time at which the X-ray intensity has decreased to 50% is denoted T = 0 and is referred to as time zero in this article.

The data were captured in single-shot mode. A large number of shots were made at the same sample position until it was permanently damaged. Measurements were performed for different sample temperatures ranging from 300 K to 500 K.

## III. DATA ANALYSIS

### A. X-ray diffraction

**FIG. 2.**
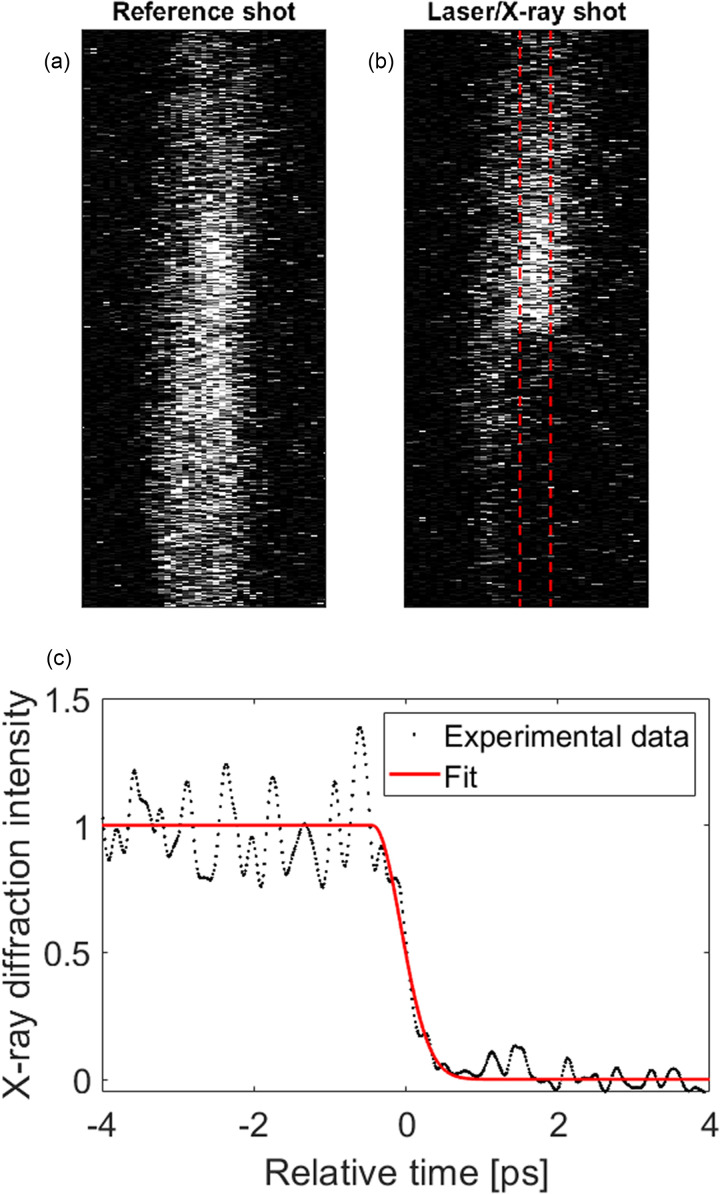
(a) and (b) show the reference shot and the shot with the laser, respectively. Time increases from the top of the images to the bottom. The red dashed lines in the laser shot indicate the part of the image used in the analysis, where the laser has a uniform fluence. (c) shows the laser shot (normalized using the reference shot) together with a Gaussian fit. Time zero is defined as the time at which the intensity has decreased to 50%. This dataset was acquired at 300 K.

In 2005, Lindenberg *et al.*^11^ showed that the time evolution of the diffracted X-ray intensity for a specific Bragg reflection could be described by

$$I\left(t\right)=I(0)e^{-\frac{q^{2}v_{i}^{2}t^{2}}{3}},\;t>0,$$ (1)

where q is the reciprocal lattice vector for the studied reflection, v_i_ is the initial velocity of the atoms upon bond-breaking, and t is time. Before each laser/X-ray shot, a reference shot was acquired without the laser, to determine how the X-ray diffraction efficiency changed over time. A Gaussian function was fitted to the measured time-dependent X-ray diffraction efficiency in order to extract disordering time and time zero. Figure 2 shows a reference and a laser/X-ray shot, with the corresponding lineout in time together with the Gaussian fit.

**FIG. 3.**
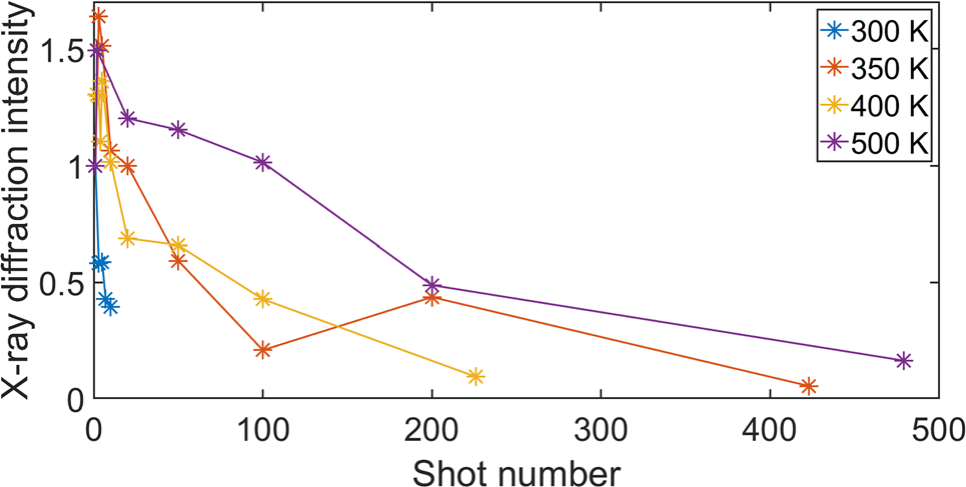
X-ray diffraction intensity of reference shots as a function of the number of shots at the different temperatures investigated. The intensity is normalized to the intensity of the first shot at each temperature.

After each laser shot, the sample resolidifies, allowing repeated measurements to be made. However, crystal regrowth is not perfect, and the X-ray diffraction efficiency degenerates, making it difficult to evaluate time zero accurately. The reference shots were used to evaluate the change in X-ray diffraction efficiency during repeated melting. The X-ray diffraction intensity is shown in Fig. 3 as a function of shot number for the different sample temperatures investigated. The intensity is normalized to the intensity of the first reference shot at each temperature. A clear difference can be seen between the measurement at 300 K and those at higher temperatures. At 300 K, the sample was destroyed after only a few shots, while at higher temperatures, it was possible to melt the sample and determine time zero of more than 200 times.

Another effect limits the number of measurements that can be performed at the same sample position above room temperature. There is an increase in the X-ray diffraction efficiency after time zero resulting from surface structures, which degrades the contrast with the initial X-ray diffraction efficiency, thus increasing the uncertainty in the determination of time zero. Laser-induced periodic surface structures (LIPPS) are well-documented in experiments using laser-exposed semiconductor materials and metals.^16,17^ Sipe *et al.* have described this phenomenon as a result of interference between the refracted laser beam and scattered fields in the material induced by surface roughness. This leads to inhomogeneous energy absorption causing periodic ripples on the surface.^18^

**FIG. 4.**
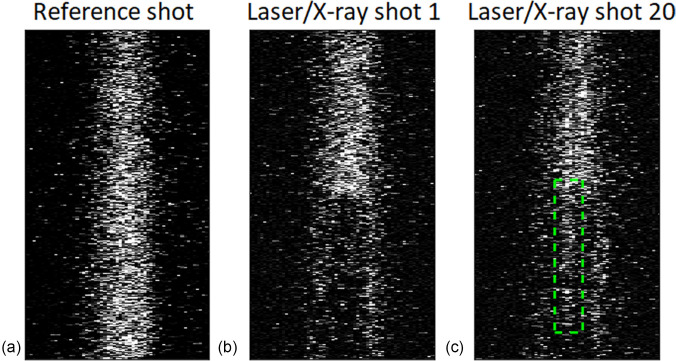
Images of (a) the reference shot, (b) the first laser/X-ray shot on the pristine sample, and (c) the twentieth laser/X-ray shot on the same position. The green dashed rectangle indicates the increase in the X-ray diffraction intensity seen in the later shots. The data were acquired at 500 K.

**FIG. 5.**
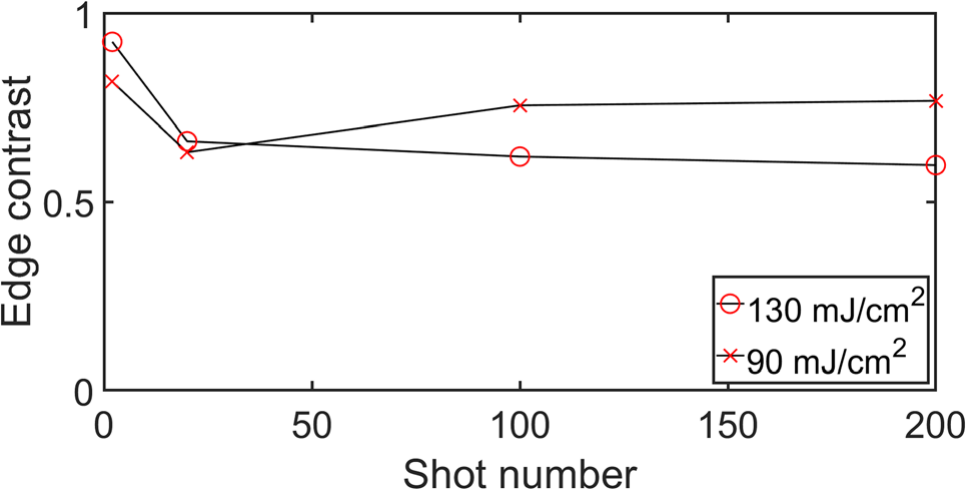
Edge contrast between the X-ray intensity before and after time zero for 130 mJ/cm^2^ and 90 mJ/cm^2^, respectively.

The effect of LIPPS on the X-ray reflectivity has been investigated by Jurgilaitis *et al.*^19^ They found that surface ripples could lead to an increase in X-ray diffraction efficiency. Upon laser irradiation, ripples are formed on the sample surface and modulate the incidence angle of the X-ray beam. A portion of the X-ray beam will impinge on the sample with a higher incidence angle, thus increasing the penetration depth.^19^ In the present work, time zero is indicated by the reduction in X-ray diffraction efficiency due to a molten layer of InSb. When the penetration depth is increased due to the formation of ripples, the X-rays will penetrate the still crystalline material to a greater extent. This effect can be seen as a deviation in the laser pump depth and X-ray probe depth overlap, making it more difficult to determine time zero. This effect becomes prominent after a few tens of shots in measurements made above room temperature and is most pronounced in the center of the X-ray footprint, as can be seen in Fig. 4. The influence of LIPPS on the time zero determination was investigated by evaluating the edge contrast. This is defined as $(I_{0}-I_{1ps})/I_{0}$, where I_0_ is the intensity before laser interaction and I_1ps_ is the intensity around 1 ps after laser excitation. Figure 5 shows how the edge contrast changes with the number of shots for two different fluences. The edge contrast is observed to decrease in the high fluence region after several laser shots, but is still high enough to allow for time zero determination for 200 shots.

## IV. BEST OPERATING CONDITIONS WITH RESPECT TO LASER FLUENCE AND SAMPLE TEMPERATURE

The laser fluence is varying with the dimension perpendicular to the timing direction. We have extracted data for two different fluences 90 mJ/cm^2^ and 130 mJ/cm^2^. Both these fluences are in the so-called inertial regime.^12^ There is no significant difference for these two fluences regarding the number of shots that can be used for a timing monitor. At even higher fluences (above 200 mJ/cm^2^), where Hillyard *et al.* found accelerated disordering,^12^ the sample is destroyed after a few shots and does not recover regardless of the sample temperature. For lower fluences (below 50 mJ/cm^2^), we obtain thermal melting, which is slow and inaccurate as a timing monitor.

We investigated the quality of the multi-shot timing monitor as a function of temperature. There is a substantial improvement with regard to the number of shots already at 350 K, but the higher the temperature, the more useful shots per data point are obtained, as shown in Fig. 3. We were limited to heating up to 500 K and did not explore higher temperatures.

## V. EVALUATION OF AN RF-CAVITY-BASED JITTER MONITOR AND BEAMLINE JITTER

An RF-cavity-based jitter monitor, inspired by an rf-based timing tool in use at LCLS, is currently being developed at the FemtoMAX beamline as a potential timing monitor. At LCLS, the RF-cavity arrival time monitor is used to measure the electron arrival time with respect to the RF phase. At FemtoMAX, two cavities are excited: one by the pulse originating from a button beam position monitor (BMP) when the electron beam passes and the other by a pulse from an ultrafast photodiode illuminated by the visible laser. The cavity signals are digitally processed in a Teledyne-Lecroy LabMaster 10 Zi oscilloscope, which has a bandwidth of 36 GHz, to determine the relative laser/X-ray arrival time. One limitation of the RF-cavity-based monitor is the jitter between two channels of the oscilloscope, which is specified as being <250 fs.

**FIG. 6.**
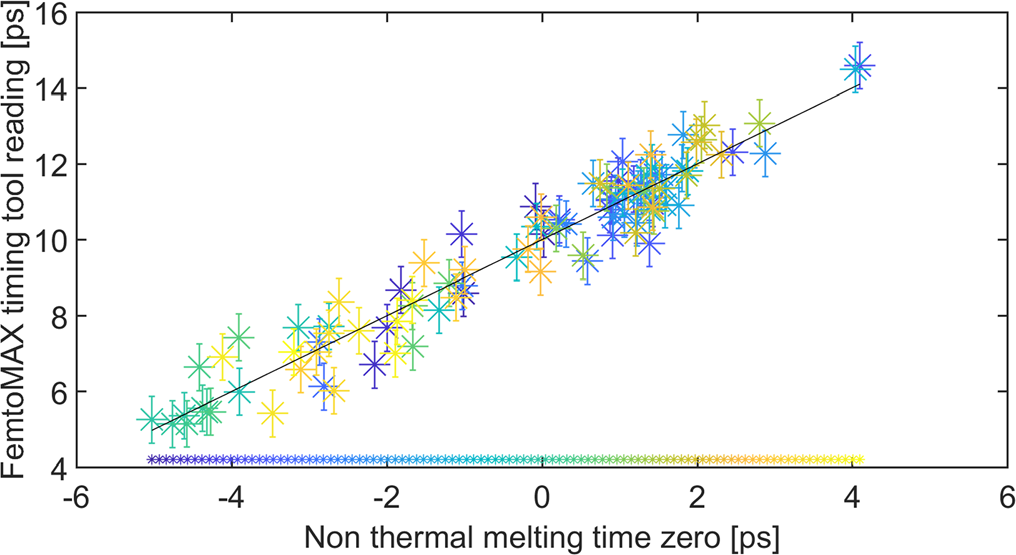
FemtoMAX timing tool reading as a function of time zero determined from non-thermal melting. The data points are color-coded according to the number of shots; the first shot is being shown in purple and the last shot in yellow. The black line is a linear fit, and the standard deviation of 600 fs is the intrinsic jitter of the RF-cavity-based jitter monitor.

The non-thermal melting setup described here was used to investigate the performance of the RF-cavity-based jitter monitor. A large number of shots were acquired at the same laser delay setting, and time zero was determined as the time at which the X-ray intensity had decreased to 50%. Figure 6 shows the RF-cavity-based jitter monitor reading as a function of the relative time zero determined with non-thermal melting. The precision of the RF-cavity-based jitter monitor is the standard deviation from the linear fit of the data, which was determined to be 600 fs. We attribute this relative value to the extraction of carriers in the photodiode. After these measurements, the rms of the RF-cavity-based jitter monitor was improved to ∼300 fs.

The data shown in Fig. 6 also contain information on the variation in timing between the laser and X-ray pulses at the beamline. For a laser/X-ray setup with no drift or jitter, the data points in Fig. 6 would all be at the same position on the X-axis. Jitter will introduce a random distribution of data points along the X-axis, whereas drift will appear as a continuous shift with an increasing number of shots. To differentiate between jitter and drift, the data points are plotted on a continuous color scale, from purple, for the first shots, to yellow, for the last shots. The clustering of certain colors at different positions on the X-axis shows that there is some drift as well as jitter at the beamline. The total variation in laser/X-ray pulse timing is approximately 8 ps.

## VI. EVALUATION OF THE ACCURACY OF THE NON-THERMAL MELTING TIMING MONITOR

**FIG. 7.**
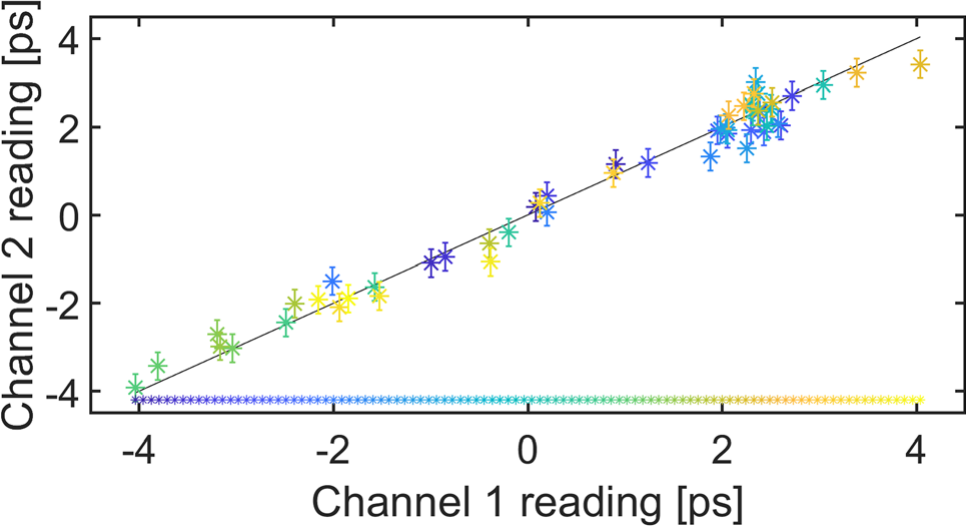
The left and right parts of the image were split into two separate channels, which were evaluated separately. This mimics the use of two identical set-ups to evaluate the performance of the timing monitor for 100 shots.

In order to evaluate the accuracy of the proposed timing monitor without a perfect timing reference, we have split our data such as the one shown in Fig. 2 into two separate channels. We have used four pixel columns to the left of the center as one channel and four pixel columns to the right of the center as a second channel. These two channels have been evaluated independently, and their correlation is shown in Fig. 7. The standard deviation of the errors is 320 fs, which contains a potential error in either channel. A single channel of the timing monitor has an accuracy, which is a factor $\sqrt{2}$ smaller that is 230 fs.

## VII. DISCUSSION

It is clear from the findings of this study that it is necessary to heat the sample above room temperature to be able to use the same sample position repeatedly, which is a requirement for the practical application of this scheme. We found that at a sample temperature of 500 K, the same sample position could be used for ∼200 measurements; however, it is possible that reducing the fluence could increase the number of measurements possible. The decrease in diffracted X-ray efficiency after repeated measurements is very likely due to the formation of imperfections during regrowth of the crystal. The superior performance of the timing monitor at 500 K, compared to 300 K, indicates that the quality of crystal regrowth is higher at higher sample temperature. Results from previous studies have indicated that repetitive melting is possible at room temperature. Navirian *et al.* performed a study in which InSb was repeatedly melted and allowed to regrow successfully on the order of 10^6^ times.^20^ However, their study was performed at a high-repetition-rate facility. This may have caused dc heating of the sample, facilitating crystal regrowth. Furthermore, the time resolution in their study was 2.5 ps, which is too long to conclusively distinguish between non-thermal and thermal melting.

**FIG. 8.**
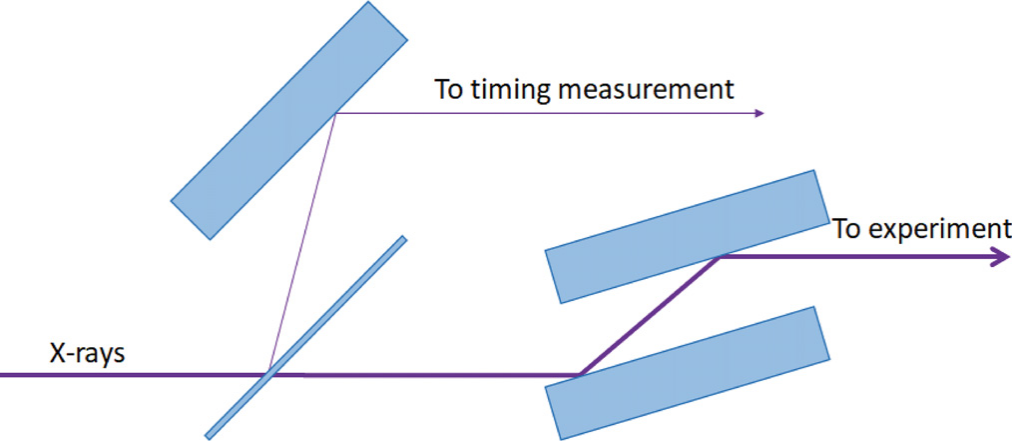
Schematic image of the InSb timing monitor setup.

A timing tool based on non-thermal melting could be permanently installed at a beamline by adding a double monochromator. The beam for timing measurements could be taken from a lower harmonic or off-center from the same harmonic as the beam used for the experiment, using thin silicon or diamond crystals. The transmission of a silicon crystal with a thickness of about 10 *μ*m is expected to be 82% at 8 keV. The set-up is shown in Fig. 8.

Recently, Epp *et al.* proposed a scheme for finding time zero based on the melting of Bi.^21^ The InSb timing monitor has several advantages. It can be mounted so that it can be inserted at the sample position and be used multiple times for verifying the time when laser and X-rays temporally overlap. The ability to give 200 shots per sample position also enables it to be used as an online timing monitor. Using a commercially available 3 inch wafer, it is possible to obtain about 1500 sample positions separated by 0.2 mm horizontally and 1 cm vertically for a single wafer, before replacement is needed. This corresponds to 300 000 shots or over 8 h of continuous data acquisition at 10 Hz. The 1 cm vertical length gives us approximately a 10 ps timing window, which presently is needed. The MAX IV Linear accelerator is still under development, and we expect jitter to go below 2 ps. In this case, the timing window could be reduced by a factor 5, and we would obtain 7500 sample points per wafer. This would allow for 4 h of data acquisition at 100 Hz. To speed up sample handling, it would be better to mount the set-up under ambient pressure rather than in a vacuum chamber. This has yet to be implemented before it is used at FemtoMAX. FemtoMAX will later go to 100 Hz.

In conclusion, we have used a non-thermal melting setup to evaluate a timing monitor currently being developed at the FemtoMAX beamline and have described how information on the jitter and drift of the beamline can be extracted. Similar schemes could be implemented at other facilities, as a beam diagnostic tool to check other instruments and for time zero determination during experiments.

## Data Availability

Raw data were generated at the MAX IV laboratory large scale facility. Derived data supporting the findings of this study are available from the corresponding author upon reasonable request.
